# Accurate and fast path computation on large urban road networks: A general approach

**DOI:** 10.1371/journal.pone.0192274

**Published:** 2018-02-14

**Authors:** Qing Song, Meng Li, Xiaolei Li

**Affiliations:** 1 School of Electrical Engineering, University of Jinan, Jinan, P. R. China; 2 School of Control Science and Engineering, Shandong University, Jinan, P. R. China; Northeast Normal University, CHINA

## Abstract

Accurate and fast path computation is essential for applications such as onboard navigation systems and traffic network routing. While a number of heuristic algorithms have been developed in the past few years for faster path queries, the accuracy of them are always far below satisfying. In this paper, we first develop an agglomerative graph partitioning method for generating high balanced traverse distance partitions, and we constitute a three-level graph model based on the graph partition scheme for structuring the urban road network. Then, we propose a new hierarchical path computation algorithm, which benefits from the hierarchical graph model and utilizes a region pruning strategy to significantly reduce the search space without compromising the accuracy. Finally, we present a detailed experimental evaluation on the real urban road network of New York City, and the experimental results demonstrate the effectiveness of the proposed approach to generate optimal fast paths and to facilitate real-time routing applications.

## Introduction

In onboard navigation systems, a primary function is to find the route from the current location of a vehicle to a desired destination with a minimum expected travel time. Vehicle’s travel time on road is usually considered to be the single variable in determining the route. However, in applications of urban road networks it is often essential to take into account the delay time at road intersections, which may be caused by traffic signals or other interference factors such as people going by. Furthermore, most times the travel time on road is trivial while comparing with the delay at road intersections, creating the possibility to use the delay time for measuring the time taken on a route.

In network theory, this corresponds to the shortest path problem, where the delay time at road intersections can be mapped as a kind of weight associated with the node. In recent years, the popular of intelligent transportation system applications have stimulated widespread research interests [1–5], yielding in various types of shortest-path computation algorithms. From the classical Dijkstra algorithm [6], which is practically far too slow for real road network applications, to the Bidirectional search [7], and the A* algorithm [8], strategies are used to reduce the search space from the initial one big circle to two smaller ones, and to an approximate ellipse in the A* algorithm. Acceleration strategies in this first stage focus on the improvement of the search strategy itself to restrict the number of nodes visited [9, 10], while using additional data of the road network such as geographical coordinates, and moreover focus on an optimization of the data structures [11].

The second stage of research focuses on very efficient path computation strategies that are practically usable in real-time onboard navigation systems. Auxiliary data are generated through a preprocessing task, and several special properties of road networks such as sparse, almost planar, and inherent hierarchical are exploited for design of high speed search techniques. Naturally, a pre-computation of the shortest paths between all node pairs would achieve extremely fast queries, however, it is unpractical for real road network applications due to its huge time and storage requirement. Plenty of research thus turned to extract and preprocess part of the helpful data that could effectively accelerate the queries [12–17]. Möhring et al. [13] partitioned the graph into regions and pre-computed a flag for each edge towards each region, indicating whether the edge lies on the shortest path to any node of this region, and the query algorithm only considered edges whose flag towards the target region was true. Maue et al. [14] pre-computed the shortest connection between all pairs of divided clusters and maintained an upper and lower bound during the query to prune the unnecessary searches.

Hierarchical strategy has proven to be very effective in reducing the search complexity of the algorithm, and there are different types of hierarchical models and heuristic search strategies for road network applications [15,16,18,19]. One makes use of the inherent hierarchical topologies of road networks [18–21], where the graph hierarchy is defined and constructed based on road categories, road lengths, speed limits, etc. The resulting path computation algorithm designed on such type of hierarchical model usually suffers from high computational errors, and it needs an assistance of a human expert to ensure the connectivity of the hierarchy as well as to improve the accuracy of path computation [18], which limits its applications. The other approach focuses more on how to partition and manage a network. The hierarchy is constructed based on the partition of the network, which are depicted in different types of models [15, 16]. The accuracy and the efficiency of the resulting path computation algorithm depends both on the model and on the search strategies. Jung and Pramanik [15] pre-computed all pairs of shortest paths between the boundary nodes of each subgraph and applied a variation of A* algorithm to accelerate the subsequent path computation. Rajagopalan et al. [16] present a sampling approach for estimating the risks of supernodes, and constituted a hierarchical abstraction graph model for fast near-optimal path computations.

In this work, we address the problem of efficient path computation on large urban road networks, where the delay time at road intersections is used for measuring the time taken on a route and can be treated as a kind of weight associated with the node. We focus on an accurate and more general approach, that is without any prior knowledge of node coordinates, road types, and without any prior knowledge of the network topology. First, we propose a new graph partitioning method with the objective of producing high balanced traverse distance partitions, and we constitute a three-level graph model based on the graph partition scheme for structuring the urban road network. Second, we develop a new hierarchical path computation algorithm that supports accurate and fast path queries in large node-weighted graphs. The algorithm benefits from the hierarchical graph model and utilizes a region pruning strategy to significantly reduce the search space without compromising the accuracy. We instantiate our approach with real-world data and study its properties, in particular the network partition effects and the performance of various heuristic algorithms, in a realistic and challenging urban environment.

The rest of the paper is organized as follows. In the next section, we introduce the basic definitions and notations used in the rest of the paper followed by a precise description of the hierarchical graph model. Further, we present the graph partitioning method. Then, we introduce the hierarchical path computation algorithm and we examine the performance of the hierarchical optimization scheme in the further section. The paper finishes with a conclusions and future directions.

## Modelling of urban road network

In this section, we present a three-level graph model for structuring the urban road network. First we give the basic definitions and notations used in the hierarchical graph model.

### Basic graph definitions

Definition 1. Given two graphs *G* = (*V*,*E*,*W*) and *G*_*u*_ = (*V*_*u*_,*E*_*u*_,*W*_*u*_) such that *V*_*u*_ ⊆ *V*, *E*_*u*_ ⊆ *E*, and *W*_*u*_ ⊆ *W*. Then, *G*_*u*_ is called the *subgraph* of *G*.

Suppose that *G* is partitioned into a set of subgraphs *G*_1_(*V*_1_,*E*_1_,*W*_1_), *G*_2_(*V*_2_,*E*_2_,*W*_2_), ⋯, *G*_*p*_(*V*_*p*_,*E*_*p*_,*W*_*p*_), then the following relation satisfies:

$$\begin{array}{l} V_{1}\cup V_{2}\cup \cdots \cup V_{p}=V,\;E_{1}\cup E_{2}\cup \cdots \cup E_{p}\subset E\\ V_{i}\cap V_{j}=\phi ,\;E_{i}\cap E_{j}=\phi ,\;where\;1\leq i,j\leq p\;and\;i\neq j. \end{array}$$

Definition 2. Given a graph *G* = (*V*,*E*,*W*), such that for any node *i*, *j* ∈ *V* there exists an edge (*i*, *j*) ∈ *E*, then *j* is called the *adjacent node* of *i*, and vice versa. The adjacent nodes set of *i* is defined by:

Adjacent(i)={j|((i,j)∈E∨(j,i)∈E)∧(i,j∈V)} (1)

Definition 3. Given a partition *P* = {*G*_1_, *G*_2_, …, *G*_*p*_} of *G*, such that for any node *i* ∈ *V*_*u*_ there exists an adjacent node *j* ∈ *Adjacent*(*i*) which satisfies *j* ∈ *V*_*v*_ and *V*_*u*_ ≠ *V*_*v*_, 1 ≤ *u*, *v* ≤ *p*. Then, *i* is called the *border node* of subgraph *G*_*u*_. The border nodes set of subgraph *G*_*u*_ is denoted by *Border*(*G*_*u*_). Subgraph *G*_*v*_ is called the *neighbor subgraph* of node *i*, denoted by *Neighbor*(*i*).

Definition 4. Given a partition *P* = {*G*_1_, *G*_2_, …, *G*_*p*_} of *G*, such that for any node *i*, *j* ∈ *V* there exists an edge (*i*, *j*) ∈ *E* which satisfies *i* ∈ *Border*(*G*_*u*_) and *j* ∈ *Border*(*G*_*v*_), 1 ≤ *u*, *v* ≤ *p* and *u* ≠ *v*. Then, edge (*i*, *j*) is called the *cut edge* between subgraphs *G*_*u*_ and *G*_*v*_. The cut edges set between *G*_*u*_ and *G*_*v*_ is defined by:

$$Cut(G_{u},G_{v})=\{(i,j)|((i,j)\in E)\wedge (i\in Border(G_{u}))\wedge (j\in Border(G_{v}))\}$$ (2)

Obviously, the cut edges separate the subgraphs to mutually disjoint sets with no overlapping nodes or edges, thus we have ∪_*u*,*v*_ *Cut*(*G*_*u*_, *G*_*v*_) = *E* – ∪_*u*_*E*_*u*_, where 1 ≤ *u*, *v* ≤ *p*, *u* ≠ *v*.

Definition 5. Given a partition *P* = {*G*_1_, *G*_2_, …, *G*_*p*_} of *G*, for any subgraph *G*_*u*_ (1 ≤ *u* ≤ *p*) the *connect edges* set within the subgraph between all border nodes is defined by:

$$Connect(G_{u})=\{(i,j)|(i,j)\in (Border(G_{u})\times Border(G_{u}))\wedge (i\overset{d_{Gu}(i,j)}{\to }j)\wedge (i\neq j)\},$$ (3)

where the weight of any connect edge (*i*, *j*) is computed by the shortest path distance from node *i* to *j* within subgraph *G*_*u*_.

Definition 6. Given a partition *P* = {*G*_1_, *G*_2_, …, *G*_*p*_} of *G*, for any subgraph *G*_*u*_ (1 ≤ *u* ≤ *p*) the *traverse distance* set of the subgraph is defined by:

$$Traverse(G_{u})=\{d_{G}(i,j)|(i,j)\in (Border(G_{u})\times Border(G_{u}))\wedge (Neighbor(i)\neq Neighbor(j))\wedge (i\neq j)\},$$ (4)

which reflects the accumulated distance increase when a shortest path passes through the subgraph *G*_*u*_. The ratio of the maximum value to the minimum one in the traverse distance set is called the *traverse distance ratio* of subgraph *G*_*u*_, denoted by *R*_*T*_(*G*_*u*_), which measures the difference in traverse distance of the subgraph.

### Graph hierarchy definitions

Definition 7. Given a urban road network, the *level-0 graph model* is defined by *G*^0^ = (*V*^0^,*E*^0^,*W*^0^), where

1. *V*^0^ is the set of nodes, with each node *i* ∈ *V*^0^ corresponding to a road intersection in the network.
2. *E*^0^ = {(*i*, *j*)|(*i*, *j* ∈ *V*^0^) ∧ (*i* ≠ *j*)} is the set of edges, with each edge corresponding to a road.
3. For any node *i* ∈ *V*^0^, its node weight *w*^0^(*i*) ∈ *W*^0^ corresponds to the average delay time of vehicles at *i* during a certain computing period of time.

Definition 8. Given a partition *P* = {*G*_1_, *G*_2_, …, *G*_*p*_} of level-0 graph *G*^0^, the *level-1 graph model* is defined by *G*^1^ = (*V*^1^,*E*^1^,*W*^1^), where

1. $V^{1}=\cup_{u=1}^{p}Border(G_{u})$.
2. *E*^1^ = (∪_*u*,*v*_ *Cut*(*G*_*u*_, *G*_*v*_))∪(∪_*u*_ *Connect*(*G*_*u*_)), 1 ≤ *u*, *v* ≤*p* and *u* ≠ *v*.
3. For any edge (*i*, *j*) ∈ *E*^1^, its edge weight

$$w^{1}(i,j)=\left\{ \begin{array}{l} d_{G_{u}}(i,j)\;(i,j)\in Connect(G_{u})\\ 0\;\;\;\;otherwise \end{array}\right..$$

Definition 9. Given a partition *P* = {*G*_1_, *G*_2_, …, *G*_*p*_} of level-0 graph *G*^0^, the *level-2 graph model* is defined by *G*^2^ = (*V*^2^,*E*^2^,*W*^2^), where

1. Each node *i* ∈ *V*^2^ corresponds to a subgraph.
2. Each edge (*i*, *j*) ∈ *E*^2^ corresponds to the collection of cut edges between the subgraphs.
3. For any node *i* ∈ *V*^2^, its node weight

$$w^{2}(i)=\min Traverse(i).$$

**Fig 1:**
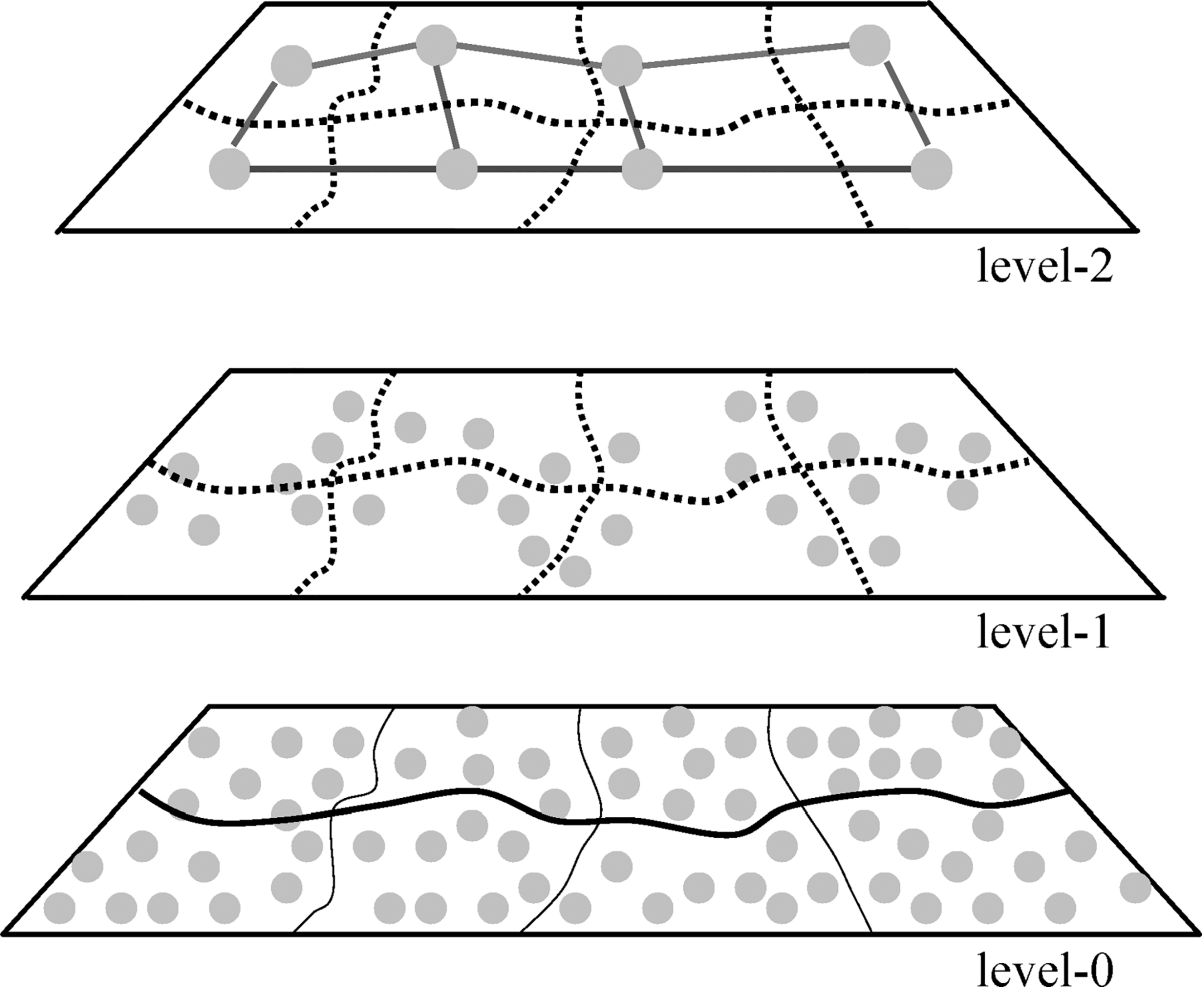
Illustration of the three-level graph model.

Fig 1 is an illustration of the model. The level-0 graph is the original urban road map that consists of all nodes and edges. A fragmentation of 8 partitions is applied on the level-0 graph, and all border nodes at this level are extracted as the nodes at the next higher level (level-1). The level-2 graph consists of 8 nodes, which is far less than the number of nodes at the original level (level-0).

## Graph partitioning algorithm

In this section, we present an agglomerative graph partitioning algorithm for producing high balanced traverse distance partitions. The basic objective is to reduce the difference in traverse distance, that is to reduce the traverse distance ratio of each subgraph, while keeping the subgraph size to a setting range [*δ*_*L*_, *δ*_*U*_].

The algorithm is composed of the following two steps:

Step 1: Initialization of subgraphs. In this step, the initial network (level-0 graph) is divided into a series of small subgraphs, where each border node has only one neighbor subgraph. This can be achieved by checking the nodes one by one: for each unassigned node *i* (all nodes are unassigned with the subgraph number in the beginning), we proceed as follows

1. Judge whether *i* has more than one adjacent node. If so, assign a new subgraph number *Num* to node *i*, and maintain a set *Sub* for the nodes assigned to this subgraph in each iteration. *Sub* = {*i*} in the beginning, then we repeat adding the unassigned adjacent nodes of the node in *Sub*, that is
  Sub={unassignedadjacentnodesofSub},
  and assign the subgraph number *Num* to the nodes in *Sub*. This process is repeated until the subgraph size of *Num* is greater than *δ*_*i*_. If *δ*_*i*_ cannot be achieved after several iterations, move all nodes of *Num* to a neighbor subgraph and reassign the subgraph number.
2. Delete the first element in *Sub*, i.e. node *j*, judge whether *j* has an adjacent node *x* whose subgraph number is not *Num*. If not, go to 3); Otherwise, add all unassigned adjacent nodes of *j* and *x* to the end of the set *Sub*, and assign them the subgraph number *Num*, and go to 4).
3. For any unassigned node *x*, judge whether *x* has an adjacent node whose subgraph number is not *Num*, add the node *x* to the end of the set *Sub* and assign it the subgraph number *Num* only if the condition holds, and then go to 4).
4. Exit if the set *Sub* becomes null; Otherwise, go to 2) and continue.

In the end, we move the unassigned degree one node (with only one adjacent node) to its neighbor subgraph and assign them with the subgraph number. At this point, all nodes are assigned to a subgraph, and each border node has only one neighbor subgraph.

Step 2: Subgraph agglomeration. In this step, an agglomeration process is performed on the decomposed small subgraphs produced in Step 1, with the objective of reducing the traverse distance ratio and regulating the subgraph size to the setting range. This can be achieved by greedily merging two neighboring subgraphs which yields the smallest traverse distance ratio, as long as the combined subgraph size is below *δ*_*U*_.

1. Traverse distance ratio computation for each subgraph. Based on the decomposed subgraphs in Step 1, we can easily construct the level-1 graph *G*^1^ by extracting the border nodes, the cut edges, and adding the connect edges between the border nodes of each subgraph. Then, a local shortest path tree is constructed from each node of *G*^1^: starting with the border node as the root, a Dijkstra search on *G*^1^ is stopped as soon as the current distance is already greater than the maximal weight of the connect edges incident to the root. Then we get the traverse distance set for each subgraph, and the traverse distance ratio can be computed by dividing the maximum value by the minimum one.
2. Heuristic agglomeration process. For each subgraph *G*_*u*_, we evaluate the traverse distance ratio decrease that would happen by merging a neighboring subgraph to *G*_*u*_. Combine *G*_*u*_ with the subgraph which yields the maximum ratio decrease, but only if the combined subgraph size is below *δ*_*U*_. This process is repeated until all subgraphs are within the size of [*δ*_*L*_, *δ*_*U*_] and no further improvements can be achieved. Note that the merging of subgraphs may simultaneously affect the traverse distance ratio of a neighboring subgraph, thus we need to update the ratio for such potentially affected subgraphs.

## Hierarchical path computation algorithm

Based on the preceding graph model, we develop a hierarchical path computation algorithm using a region pruning strategy (HiARP) for accurate and fast path computations. We mainly discuss the case that the source and destination nodes are far away from each other; for the case that the source and destination are within the same subgraph or in adjacent subgraphs, we turn to compute the shortest path directly using the level-0 graph.

### HiARP algorithm

The HiARP algorithm generally follows the Dijkstra search, where the search first starts on the level-2 graph for an estimation of the shortest path length between source node *s* and destination node *d*, then the search switches to the level-0 graph, starting from the source node *s*, and once the border nodes are reached, the search will switch to the next higher level (level-1). A close upper bound on the shortest path length between *s* and *d* is maintained and tightened repeatedly during the search. Whenever the search on level-1 reaches a subgraph, a lower estimate will be calculated and the whole subgraph region will be pruned if this lower estimate exceeds the upper bound.

The algorithm is composed of the following steps:

Step 1: Search on *G*^2^. Identify the source subgraph *G*_*s*_ which contains the source node *s* and the destination subgraph *G*_*d*_ which contains the destination node *d*. Initialize a distance value *d*_2_(*G*_*u*_, *G*_*d*_) for every subgraph *G*_*u*_, set it to zero for the destination subgraph *G*_*d*_ and infinity for all the other subgraphs. Then start search from *G*_*d*_ on the level-2 graph *G*^2^ following a standard Dijkstra search, until all subgraphs are settled. Suppose that the shortest path between the destination subgraph *G*_*d*_ and the source subgraph *G*_*s*_ is

$$SP_{2}(G_{d},G_{s})=<G_{d}(G_{0})\to G_{1}\to \cdots \to G_{s}(G_{k})>.$$ (5)

**Fig 2:**
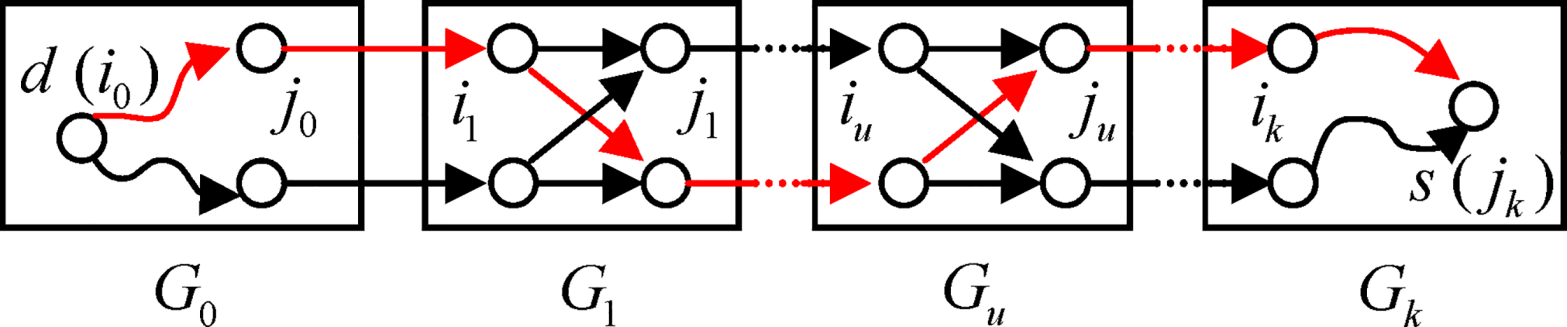
Path computation within the subgraph region of *SP*_2_(*G*_*d*_,*G*_*s*_).

To compute an upper bound on the shortest path length from subgraph *G*_*u*_ (*G*_*u*_ ∈ *SP*_2_(*G*_*d*_, *G*_*s*_)) to the destination *d*, we utilizes the level-1 graph, and extracts the nodes and edges within the subgraph area of *SP*_2_(*G*_*d*_, *G*_*s*_) and extracts the cut edges between these subgraphs. Add an edge from *d* to every border node *j*_0_ (*j*_0_ ∈ *Border*(*G*_*d*_)), where the weight of the edge is assigned with the maximum weight of the connect edges incident to *j*_0_, and the same edge adding process from the border node *i*_*k*_ (*i*_*k*_ ∈ *Border*(*G*_*s*_)) to the source *s*. Then the shortest path from *d* to *s* within this constructed area can be easily obtained, as shown in Fig 2, where the shortest one is denoted by red lines. Let $\hat{d}(i,d)$ denote the path length from *d* to any node *i* on the shortest path. Based on this practical route, we get an upper bound on the shortest path length from every subgraph *G*_*u*_ ∈ *SP*_2_(*G*_*d*_, *G*_*s*_) to *d*, and from the source *s* to *d*, that is:

$$Upper_{G_{u}\to d}=\hat{d}(j_{u},d),u=0,1,\cdots ,k$$ (6)

$$Upper_{s\to d}=\hat{d}(s,d)$$ (7)

Step 2: Search on (*G*^0^, *G*^1^). Initialize a distance value *d*(*s*, *i*) for every node *i* ∈ *V*^0^, set it to *w*^0^(*s*) for the source node *s* and infinity for all the other nodes. Record the predecessor node *P*(*i*), which preceeds *i* in an optimal shortest path from the source. Set *P*(*s*) = *s* for the source *s* and null for the other nodes. Then mark the source *s* as the current node and start the search.

1. Judge whether the current node *i* (*i* = *s* in the beginning) is in the level-0 graph *G*^0^. If not, go to 2); Otherwise, relax the adjacent node *j* of *i* on *G*^0^, which amounts to replace *d*(*s*, *j*) with a new value (*d*(*s*, *i*) + *w*^0^(*j*)), but only if this value is smaller. Overwrite the predecessor of *j* to *P*(*j*) = *i* if the distance to *j* is updated, and then go to 4).
2. Judge whether the current node *i* and the predecessor *P*(*i*) are in the same subgraph. If not, go to 3); Otherwise, compute the lower bound on the shortest path length from *s* to *d* via subgraph *Neighbor*(*i*), that is
  $$Lower_{s\to d}=d(s,i)+d_{2}(Neighbor(i),G_{d})$$ (8)
  The region pruning condition will act only if this lower estimate exceeds the current upper bound *Upper*_*s*→*d*_. If the condition satisfies, mark the subgraph *Neighbor*(*i*) as pruned and go to 4), and since then all searches growing into that subgraph region will be pruned; Otherwise, relax the cut edge (*i*, *j*) ∈ *E*^1^, which amounts to replace the distance to *j* by (*d*(*s*, *i*) + *w*^0^(*j*)) if this yields a smaller value. Update the predecessor of *j*, and then go to 4).
3. Tighten the upper bound *Upper*_*s*→*d*_ if *i* is a border node of subgraph *G*_*u*_ ∈ *SP*_2_(*G*_*d*_, *G*_*s*_) (1≤*u*≤*k*):
  $$Upper_{s\to d}=\min\{Upper_{s\to d},(d(s,P(i))+w^{1}(i,i_{u})+Upper_{G_{u-1}\to d})\}$$ (9)
  Relax the connect edges incident to *i*: for each endpoint *j*, replace the distance to *j* by (*d*(*s*, *i*) + *w*^1^(*i*, *j*) – *w*^0^(*i*)) if this yields a smaller value. Update the predecessor of *j*, and go to 4).
4. Extract the node *i* with the minimum *d*(*s*, *i*) value from all the unmarked nodes. Exit if the node *i* = *d*; Otherwise, mark the node *i* as current, and go to 1) and continue.

At the end of the algorithm, we get the shortest path length *d*(*s*, *d*) from *s* to *d*, and the path can be concatenated through the record of predecessor nodes. The HiARP algorithm can be used efficiently to address the path computation problem for very large node-weighted graphs. The hierarchical strategy decomposes the complex search problem into several light searches on the graph hierarchies, and the region pruning strategy greatly reduces the search space by eliminating unnecessary subgraph regions, moreover instead of relaxing all adjacent nodes, the relaxation operation in Step 2 is also simplified, which makes HiARP much faster than classical hierarchical path computation algorithms, while the path computed by HiARP is optimal.

### Optimality of HiARP

We give a theoretical proof for the optimality of the HiARP algorithm from the following two aspects. First, it is natural that the upper bound will never underestimate the optimal shortest path length, and the lower bound will never overestimate that. Thus, for any pruned subgraph region *Neighbor*(*i*), the shortest path will certainly bypass *i* due to the fact that *Lower*_*s*→*d*_ > *Upper*_*s*→*d*_ > *d*(*s*, *d*), hence there is no need to relax the edge incident to *i*, which can never appear on the shortest path; as the region pruning condition holds thereafter, all search branches growing into the subgraph region later will be pruned, and this will not affect the optimality of the algorithm. Second, the relaxation operation in Step 2 will not affect the nature of optimality in a Dijkstra’s search. In Step 2–1), we perform relaxation for all nodes adjacent to the current node on *G*^0^, which is equivalent to a standard Dijkstra’s search. Step 2–2) and 3) denote a hierarchical search on the level-1 graph *G*^1^, where in 3) we also relax all edges incident to the current node; while in 2) we only perform relaxation on the cut edge incident to the current node *i*. There is no need to relax the connect edges (*i*, *j*′) ∈ *E*^1^ since *i* and *P*(*i*) are located in the same subgraph, thus *w*^1^(*P*(*i*), *j*′) already gives the optimal path length for the connect edges (*P*(*i*), *j*′) ∈ *E*^1^, and any relaxation towards connect edges just equals to a weight modification inside the subgraph, which has already been done by the relaxation operation at *P*(*i*). Therefore, we have proven the optimality of the HiARP algorithm. □

## Experimental evaluation

To verify the validity of our approach, we consider the real urban road network of New York City (freely available data from [22]). The network is transformed to a graph with 366923 nodes and 1557956 edges, where nodes and edges correspond to the roads and the intersecting points of roads respectively, and the travel time data is used to simulate the average delay time of vehicles at nodes. We present numerical evaluations of HiARP algorithm using different graph partitions, compared to the well-known HIPLA algorithm [16], and hierarchical Dijkstra algorithm in order to analyze the main influencing factors on the algorithm performance. Also, we study the performance of various algorithms in terms of computational time and accuracy in order to verify the effectiveness of the proposed heuristics to real-time routing applications.

### Settings

Four partition schemes are employed in our testing, which are generated at different stages of our graph partitioning algorithm, as shown in Table 1. The following parameters are counted:

**Table 1:** Graph partition schemes used in testing.

Partition Schemes	*p*	${\bar{R}}_{T}$	*R*_*N*_	*|V*^1^*|*	*|E*^1^*|*
Scheme 1	2543	8.36	134.44	43282	1068699
Scheme 2	1712	9.11	134.44	36240	971335
Scheme 3	894	9.31	167.07	22960	789790
Scheme 4	510	13.64	68.53	19115	761172

- *p*: the number of subgraphs in a graph partition
- ${\bar{R}}_{T}$: the average traverse distance ratio of a graph partition, defined as

$${\bar{R}}_{T}=\frac{1}{p}\sum_{u=1}^{p}\frac{\max Traverse(G_{u})}{\min Traverse(G_{u})}$$

- *R*_*N*_: the ratio of maximum number of nodes in the subgraph to the minimum one (which measures the difference in subgraph size)
- |*V*^1^|: the number of nodes in the level-1 graph
- |*E*^1^|: the number of edges in the level-1 graph

Generally, the average traverse distance ratio ${\bar{R}}_{T}$ follows a downward trend with the agglomeration process of subgraphs, though it may fluctuate slightly. Here we select the partition scheme with a tentative rising ${\bar{R}}_{T}$ value to facilitate the performance analysis and comparison of the query algorithm. As we can see from Table 1, the level-1 graph contains fewer nodes and edges as the subgraph agglomerates, the number of nodes drops, and the average degree increases slightly.

For each partition scheme, we conduct five tests each using the HiARP, the HIPLA, and the hierarchical Dijkstra algorithm as the search rule. Each test is made to solve a set of 200 route requests using the same randomly generated source and destination nodes. It should be pointed out that during the execution of HIPLA, we employ some pre-computed data to facilitate the path retrieval within subgraphs, which makes it much faster than the initial method. All the algorithms are implemented in Matlab 7.8.0 on an Intel Xeon X5482 Dual Core processor with 32GB of RAM and the system ran Microsoft Windows Vista.

### Performance analysis

**Fig 3:**
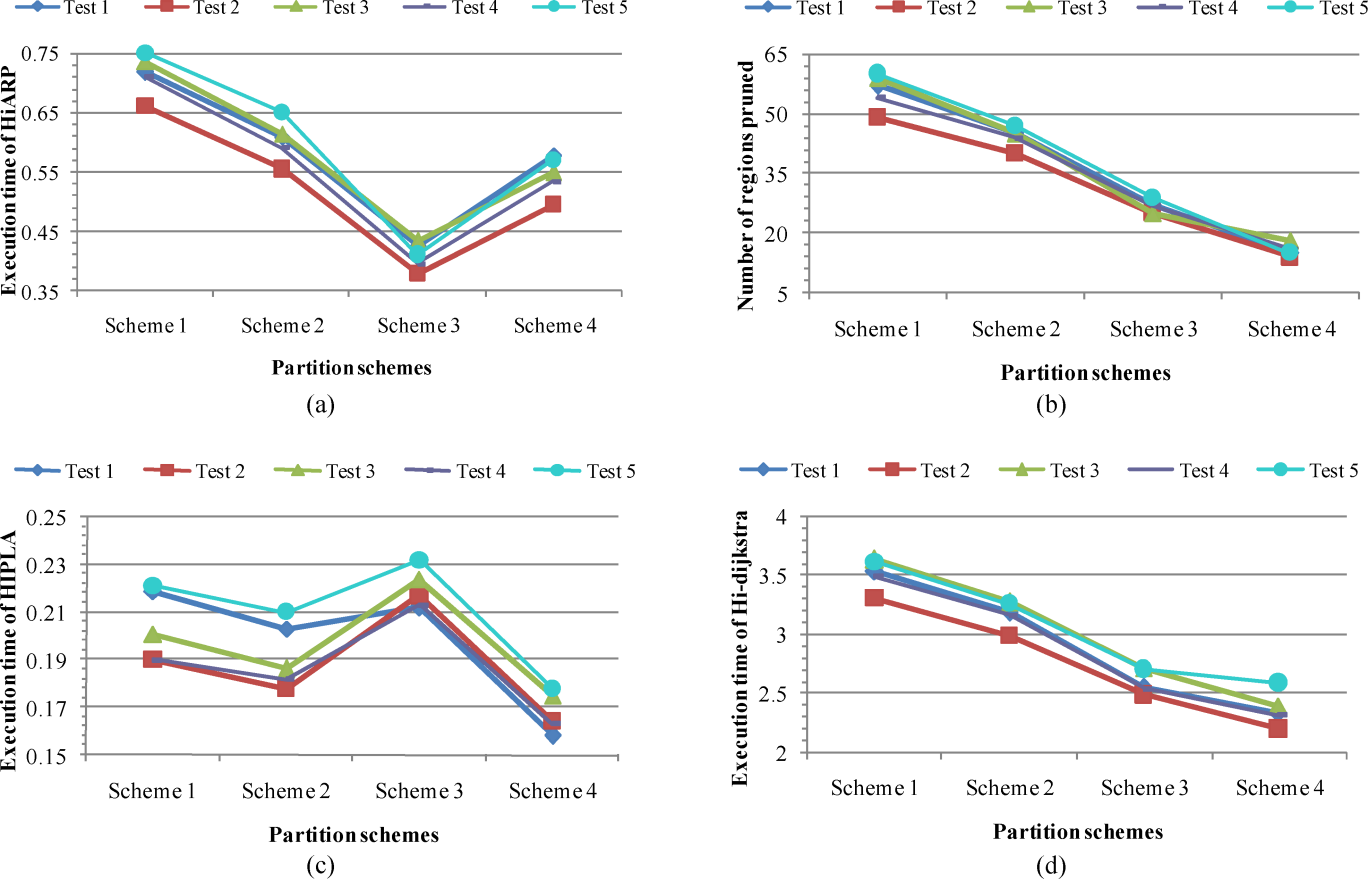
Computational costs comparison of various algorithms on Schemes 1–4. (a) Average execution time of HiARP for the five test sets. (b) Average number of regions pruned during the search of HiARP. (c) Average execution time of HIPLA for the five test sets. (d) Average execution time of hierarchical Dijkstra algorithm for the five test sets.

Fig 3 shows the average computational costs of HiARP, HIPLA, and hierarchical Dijkstra algorithm (Hi-dijkstra) under Schemes 1 to 4, where the numbers presented are average values over 200 route requests. Here, similar trends are observed in the tests of each algorithm.

In Fig 3A, the execution time of HiARP algorithm reaches a minimum value in Scheme 3. It is noted that the efficiency of HiARP is affected simultaneously by the average traverse distance ratio ${\bar{R}}_{T}$ and by the number of subgraphs *p*. Naturally, the efficiency of a hierarchical routing algorithm will be enhanced with a decreasing number of subgraphs, as the corresponding level-1 graph contains fewer nodes and edges. Thus, the execution time of HiARP drops in Schemes 1 to 3 when the increase in ${\bar{R}}_{T}$ has not become a leading factor. However, when ${\bar{R}}_{T}$ exceeds a certain value, the efficiency of HiARP will be greatly weakened since the increase in ${\bar{R}}_{T}$ leads to an even larger increase in the search space, compared to the size reduce of the level-1 graph. Hence, the execution time of HiARP increases in Scheme 4 though its number of subgraphs is the smallest.

In Fig 3B, we count another variable, the average number of subgraph regions that are pruned during the search, which depicts the efficiency of HiARP from another point of view. We observe that the number of regions pruned drops as *p* reduces in Schemes 1 to 4. Generally, the more regions pruned at an early search stage, the more efficient the HiARP would be. Thus, the number of regions pruned can only reflect the efficiency of HiARP to some extent, whereas the stage when the region is pruned is more important.

In Fig 3C, we find a similar fluctuation on the execution time of HIPLA. The efficiency of HIPLA is generally improved with the decrease of *p*, as the corresponding abstraction graph [16] contains fewer nodes and edges, which leads to the downward trend in Schemes 1 to 2. Moreover, the balance in subgraph size can also affect the query efficiency, as HIPLA will search routes within the subgraph of path *SP* [16]. Hence, the execution time of HIPLA increases in Scheme 3 due to the rising ratio of *R*_*N*_, and then drops in Scheme 4 as a result of the decreasing *R*_*N*_ and *p*.

In Fig 3D, the execution time of hierarchical Dijkstra algorithm decreases in all the five tests. It is reasonable that the efficiency of hierarchical Dijkstra algorithm depends particularly on the graph size, though the average degree of nodes may also affect it more or less. With a dropping number of subgraphs, the constructed level-1 graph contains fewer nodes and edges, thereby leading to a decreasing trend on the execution time over Schemes 1 to 4.

### Performance comparison

Table 2 shows the average computational time and accuracy of HiARP, HIPLA, and hierarchical Dijkstra algorithm under Schemes 1–4, where the numbers presented are average values over all five test sets (an average of 1000 route requests to be precise). We observe that the execution time of HiARP is much less than that of hierarchical Dijkstra algorithm. As we can see the average execution time of HiARP on Scheme 1 is 0.72s, whereas the average execution time of hierarchical Dijkstra algorithm is 3.51s. Both HiARP and hierarchical Dijkstra algorithm compute the accurate optimal path for all route requests, which is consistent with our theoretical predictions in the Optimality of HiARP Section. We also notice that the HIPLA achieves the best time efficiency in all partition schemes, e.g., the average execution time of HIPLA on Scheme 1 is 0.20s; however, large errors are observed in its path computation results based on our testing graph. The average error observed in HIPLA is around 37.68%, with a maximum error of up to 242.5%, which is inapplicable to high-precision routing applications.

**Table 2:** Computational times and accuracy comparisons of various algorithms on Schemes 1–4.

Algorithm	Scheme 1			Scheme 2			Scheme 3			Scheme 4		
	*t*	$\bar{E}$%	*E*_*max*_ %	*t*	$\bar{E}$%	*E*_*max*_ %	*t*	$\bar{E}$%	*E*_*max*_ %	*t*	$\bar{E}$%	*E*_*max*_ %
Hi-dijkstra	3.51	0	0	3.17	0	0	2.60	0	0	2.36	0	0
HiARP	0.72	0	0	0.60	0	0	0.41	0	0	0.55	0	0
HIPLA	0.20	38.16	154.57	0.19	40.13	242.50	0.22	36.09	164.36	0.17	36.34	164.48

*t* represents the average computational time (in seconds); $\bar{E}$ and *E*_*max*_ represent the average and maximum computational errors.

To further illustrate the performance improvements, we give a theoretical analysis on the computational complexity of HiARP, compared to the hierarchical Dijkstra algorithm and HIPLA. In a network with |*V*^0^| nodes and *p* subgraphs, the average number of nodes in each subgraph is *λ* = |*V*^0^|/*p*. As the Dijkstra's search runs in O(|*V*^0^|log|*V*^0^|) time for sparse networks, the computational complexity of HIPLA is O(*p*log*p*)+O(*λ*log*λ*). Suppose that the level-1 graph contains |*V*^1^| nodes and |*E*^1^| edges, thereby the average number of border nodes in each subgraph is *b* = |*V*^1^|/*p*, and the path computational cost of hierarchical Dijkstra algorithm is of complexity O(|*V*^1^|log|*V*^1^|)+O(*λ*log*λ*). In our HiARP algorithm, the path computation is broken into two subprocesses, that is path search over the level-2 graph *G*^2^ with *p* nodes which incurs O(*p*log*p*) time, and path search over (*G*^0^, *G*^1^) which incurs O(*λ*log*λ*)+O(*kb*log(*kb*)) time, where *k* is the average number of subgraph regions visited on the level-2 graph. Thus, the computational complexity of HiARP is O(*p*log*p*)+O(*λ*log*λ*)+O(*kb*log(*kb*)). For a well-partitioned graph which gives a good initial heuristics on the region pruning, *k* is quite small compared to *p*, specifically when *k*<max{*p*/*b*, *λ*/*b*}, the complexity of HiARP is approximately O(*p*log*p*)+O(*λ*log*λ*), which equals to that of the HIPLA. In this case, the HiARP will search routes only within the subgraph area of *SP*_2_(*G*_*d*_, *G*_*s*_), and all the other subgraphs will be pruned as a result of the region pruning condition. While on the contrary when the graph is not so well-partitioned, i.e., with large value of ${\bar{R}}_{T}$, few subgraph regions will be pruned during the execution of HiARP and the computational complexity will approximate to that of the hierarchical Dijkstra algorithm. This case can not happen anymore under our partition scheme, as the region pruning condition will always act since *kb*<<|*V*^1^|. Therefore, the complexity of HiARP is much lower than that of the hierarchical Dijkstra algorithm and time efficiency is greatly improved.

## Conclusions

In this paper, we have addressed the problem of efficient path computation on large urban road networks. We have developed a new graph partitioning method that can help significantly reduce the search space and thereby accelerate the path computation. By modelling the road network in a three-level graph structure, we have decomposed the complex search problem into several light searches on the graph hierarchies. We have then proposed a new hierarchical path computation algorithm HiARP using a region pruning strategy, and we have formally proven that the shortest path computed by HiARP is optimal. Our HiARP algorithm is computationally very efficient and is readily applicable for path computation problems for very large node-weighted graphs. We have instantiated our approach with real-world data to study the algorithm performance under different graph partition schemes, compared with two famous algorithms viz., HIPLA and hierarchical Dijkstra algorithm, both theoretically and experimentally. The experimental evaluation has confirmed the effectiveness of the proposed graph partitioning and path computation algorithms to generate optimal fast paths so as to facilitate real-time routing applications.

The hierarchical model framework proposed in this paper including the graph partitioning and the hierarchical path computation algorithm provides a general approach for accurate optimal path computation on large node-weighted graphs, and this can be easily extended to edge-weighted graphs. Thus, as part of future research, it would be beneficial to see the application of the proposed approach to other types of networks, e.g., social networks, communication networks. Also, it is worth quantifying the influences of the number of subgraphs and the average traverse distance ratio on the query times, in order to determine the optimum value regions. And more complicated traffic conditions can be added to the model, and we will further study the algorithm performance on such networks.

## Supporting information

S1 Code Source code of the HiARP algorithm. (ZIP)
